# Behavioral Smartphone Orientation Is Associated with Upper Limb Dysfunction Among University Students: A Cross-Sectional Study

**DOI:** 10.3390/jcm15207857

**Published:** 2026-10-12

**Authors:** Iga Szukalska, Agnieszka Kempinska-Podhorodecka, Małgorzata Szczuko

**Affiliations:** 1 Department of Bromatology and Nutritional Diagnostics, Pomeranian Medical University, 70-111 Szczecin, Poland; 2 Department of Functional Anatomy, Pomeranian Medical University in Szczecin, 70-111 Szczecin, Poland; agnieszka.kempinska.podhorodecka@pum.edu.pl

**Keywords:** smartphone, upper limb, hand grip strength, pain, Quick DASH, NRS

## Abstract

**Background:** Excessive smartphone use has been associated with musculoskeletal disorders; however, the relationship between smartphone orientation and objective measures of upper limb function remains insufficiently explored. The aim of this study was to determine whether smartphone orientation is associated with upper limb disability, grip strength, pain intensity and clinical signs of musculoskeletal dysfunction in university students. **Materials and methods:** The study involved 84 students including women (*n* = 74) and men (*n* = 10), with an age of 21 ± 2.6 years. This cross-sectional study utilized the Smartphone Orientation Scale (SOS) to assess behavioral smartphone orientation. Functional disability was assessed using the QuickDASH questionnaire and the intensity of pain was measured using the Numeric Rating Scale (NRS). Objective assessments included hand grip strength measurements via an electronic dynamometer and clinical evaluation using the Finkelstein test. Because the analyzed variables did not follow a normal distribution, Spearman’s rank correlation analysis was performed to assess the relationship between variables across the study group with statistical significance set at *p* < 0.05. **Results:** Higher SOS scores were associated with lower left- and right-hand grip strength (ρ = −0.391 and −0.395, respectively; Holm-adjusted *p* = 0.001 for both) and greater upper-limb disability measured by QuickDASH (ρ = 0.387; Holm-adjusted *p* = 0.001). SOS score was also associated with a positive right Finkelstein test (ρ = 0.294; Holm-adjusted *p* = 0.020), but not with the left Finkelstein test. Longer daily smartphone-use duration was associated with lower grip strength and higher SOS and QuickDASH scores after Holm correction. **Conclusions:** Screening using the Smartphone Orientation Scale may help identify young adults presenting with behavioral patterns associated with upper limb dysfunction. Smartphone orientation may represent a more clinically relevant behavioral marker of early upper limb dysfunction than self-reported daily smartphone use.

## 1. Introduction

Three major dimensions of problematic smartphone use have been identified: harmful use patterns (e.g., using a phone while driving), anti-social use (e.g., unauthorized use) and addictive use characterized by symptoms of dependence [1,2]. A phenomenon closely associated with problematic smartphone use is nomophobia, defined as the anxiety or fear experienced when individuals are unable to access or use their mobile phones. Symptoms of nomophobia resemble those observed in anxiety disorders and include hyperventilation, tachycardia, disorientation and sweating [3]. People affected by nomophobia try to avoid places where smartphone use is prohibited. They become inseparable from their charger or carry a second, spare phone. Nomophobia most commonly affects young adults aged 20–24, with women being particularly at risk [4]. Children are being introduced to mobile devices at increasingly younger ages. Even infants and toddlers aged 6–36 months are increasingly exposed to smartphones. A worrying fact is the amount of screen time among children under the age of two, which averages around 2 h a day [5]. According to the 2024 Global Digital Overview Report, the average daily screen time exceeds 6 h and 40 min worldwide. In some countries, including Brazil and the Philippines, it exceeds 9 h per day [6]. Although prolonged smartphone use has been linked to musculoskeletal complaints, relatively few studies have simultaneously evaluated behavioral smartphone orientation, objective grip strength, functional disability and clinical provocative tests within the same population. Previous studies have demonstrated an association between problematic smartphone use and musculoskeletal symptoms among university students. In a large study involving more than 2000 students, smartphone addiction was significantly associated with upper-body musculoskeletal symptoms [7]. Another study involving 165 university students reported an association between smartphone addiction and DASH scores, while upper-limb pain was also associated with functional disability. They reported an association between smartphone addiction and DASH scores, while upper-limb pain was also associated with functional disability [8].

The manner in which a smartphone is held during use plays an important role in the development of musculoskeletal symptoms. Using a single finger to operate the touchscreen has been associated with an increased risk of musculoskeletal disorders, particularly affecting the wrist and fingers [9]. Holding the device with both hands reduces the activity of the extensor digitorum muscle. Typing speed during smartphone use is also relevant, and the way the user operates the screen is significant. Higher typing speeds are associated with increased activity of the extensor digitorum muscle or the abductor pollicis longus muscle [10]. Importantly, evidence suggests that musculoskeletal consequences may depend not only on the duration of smartphone use but also on specific patterns of interaction. More frequent screen unlocking was significantly associated with clinical symptoms of de Quervain’s tenosynovitis in the dominant hand [11]. However, most previous studies have focused on smartphone addiction, total daily exposure or upper limb function. It remains unclear whether smartphone orientation is associated with functional disability, pain, grip strength and clinical signs of dysfunction beyond the effects of daily smartphone use duration. The aim of this study was to investigate the association between smartphone orientation and upper limb function, hand grip strength, pain intensity and clinical signs of musculoskeletal dysfunction among university students. It is hypothesized that higher behavioral smartphone orientation scores are significantly associated with hand grip strength, increased functional upper limb disability, higher pain intensity and a greater prevalence of a positive clinical Finkelstein test among university students.

## 2. Materials and Methods

### 2.1. Participants

This cross-sectional study involved 84 students from the Faculty of Health Sciences at the Pomeranian Medical University in Szczecin. The inclusion criteria for the study were being aged 18 or over, being an active student at the Faculty of Health Sciences of the Pomeranian Medical University in Szczecin, and giving voluntary and informed consent to participate in the study. The study included 84 students aged between 18 and 28 years (mean age 21 ± 2.6 years). The study population comprised 74 women (88.1%) and 10 men (11.9%). The participants were students at the Pomeranian Medical University in Szczecin, studying physiotherapy, health psychology, clinical dietetics, nursing and clinical speech and language therapy with occupational therapy. Most participants had normal body weight (74%), while 12% were overweight and 11% were underweight, and the smallest group were those who were obese (3%). Anthropometric measurements for women showed an average width of the right and left hands of 7.4 cm and a length of 18.2 cm. For men, the mean hand width and length were 8.4 cm and 20.4 cm, respectively. The vast majority of respondents (93%) stated that they were right-handed. The remaining 7% were left-handed, whilst 2% stated that they were ambidextrous.

### 2.2. Procedures

Data collection took place at the Faculty of Health Sciences at the Pomeranian Medical University in Szczecin. In the first part of the study, participants completed a questionnaire. In total, it contained 32 questions, of which 5 were open-ended and the remainder were closed-ended. At the beginning of the questionnaire, the questions concerned socio-demographic information; this was followed by 8 closed-ended questions from the SOS questionnaire, and the final 11 closed-ended questions were taken from the Quick DASH questionnaire [12].

The Smartphone Orientation Scale (SOS) questionnaire was used to assess users’ overall smartphone orientation. It consists of 8 closed-ended questions. The questionnaire scores range from 8 to 56 points. A higher score indicates a higher level of smartphone orientation. The Quick DASH questionnaire was used to assess limitations in upper limb activity and participation, as well as pain symptoms in participants’ daily lives. It consists of 11 closed-ended questions. The questionnaire score ranges from 0 to 100. A score of 0 indicates good functionality, whilst a score of 100 indicates poor functionality. The 11-point Numeric Rating Scale (NRS) (0—no pain, 10—most severe pain) was used to assess pain [13].

### 2.3. Measurements

Hand grip strength was measured using an electronic hand dynamometer (Zhongshan Camry Electronic Co., model: EH101, Zhongshan, Guangdong, China). Measurements were taken for both the dominant and non-dominant hand. Students were positioned according to standardized ergonomic guidelines with the elbow flexed at 90 degrees. The students were seated, and the measurement began with the dominant hand. Three maximal isometric contractions were performed for each hand, with a standardized 30-s rest interval between trials. Each contraction was maintained for 3 s. Verbal encouragement was provided during each trial to ensure maximal effort. The mean value of three measurements was calculated and used for statistical analysis [14]. The final stage of the examination was the Finkelstein test. Subjects were asked to clench their hand into a fist with the thumb tucked inside; the examiner then passively flexed the clenched fist towards the elbow while stabilizing the forearm. The criterion for a positive test result was pain in the area of the styloid process of the radius, whilst the criterion for a negative result was the absence of pain [15]. Questionnaires and physical measurements were administered uniformly to all participants by trained researchers during scheduled academic sessions.

### 2.4. Statistical Analysis

Statistical analyses were performed using StatView version 5.0 (SAS Institute, Cary, NC, USA), with additional statistical analyses, including bootstrap confidence intervals, Holm’s multiple-comparison correction, and sensitivity analysis, performed using Python version 3.14.8 with the SciPy version 1.18.1 and statsmodels version 0.15.1 packages. Quantitative variables were summarized using means and standard deviations and, where appropriate, medians and ranges; categorical variables were summarized as counts and percentages. Distributional characteristics were assessed using the Shapiro–Wilk test. Because several study variables were non-normally distributed or ordinal, associations were evaluated using Spearman’s rank correlation coefficient (ρ). Daily smartphone use was treated as an ordinal exposure with five ordered categories (≤2 h/day, 2–4 h/day, 4–6 h/day, 6–8 h/day, and >8 h/day). For each association, the sample size (*n*), Spearman’s ρ, two-sided *p*-value, and 95% confidence interval were reported; confidence intervals were obtained using non-parametric bootstrap resampling with 5000 repetitions. To control the family-wise type I error arising from multiple testing, Holm’s step-down correction was applied within each prespecified family of associations. Analyses were performed for the entire sample and, secondarily, after stratification by sex. Owing to the small male subgroup (*n* = 10), sex-stratified analyses were considered exploratory. Statistical significance was defined as a Holm-adjusted *p*-value < 0.05. No a priori sample-size calculation was performed; the available sample comprised 84 eligible participants. A sensitivity calculation indicated that N = 84 provides approximately 80% power at a two-sided α = 0.05 to detect a correlation of approximately |ρ| = 0.30; smaller associations may therefore have been missed.

## 3. Results

The largest proportion of respondents reported an average daily smartphone usage of 4–6 h (39%), followed by 2–4 h per day (30), 6–8 h per day (23%), more than 8 h per day (7%) and up to 2 h per day (1%). Students reported starting to use a mobile phone regularly at an average age of 11 (median 12). The youngest users were 6 years old, and the oldest was 16. In total, 32.1% of respondents reported pain in the hand and/or wrist, whilst the remaining 67.9% had no pain. The largest proportion of respondents reported pain in the right upper limb (76%), whilst fewer reported pain in the left upper limb (14%) and in both arms (10%). Pain scores on the NRS scale were 2.2 ± 1.8 for the entire study group; the average was higher for women, at 2.3 ± 1.8, whilst for men it was 1 ± 1.2 (Table 1).

The average score on the SOS questionnaire amongst the study group (*n* = 84) was 29.1, with a median of 28. Amongst women, the average score was 29, with a median of 28. Amongst men, however, the average was 29.5, with a median of 27.5. According to the study group’s interpretation, the results indicate a high level of smartphone orientation among 56% of respondents, a low level of smartphone orientation among 32% of respondents, and an average level of smartphone orientation among 13% of respondents (Table 1).

In the Quick DASH questionnaire, the mean score for the study group was 15.7 and the median was 10.2. The mean for women was 16.7 and the median was 11.4. In contrast, the mean for men was 8.2 and the median was 3.4. Across the entire study group, the average grip strength was 22.1 kg for the left hand and 22.6 kg for the right hand. In the group of women, the average was lower than in the entire study group, at 20.7 kg for the left hand and 21.3 kg for the right hand. Men recorded higher grip strength results than women. The average for the left hand was 32.6 kg and 32.8 kg for the right hand (Table 1).

A positive result in the Finkelstein test for the left upper limb was recorded in 10% of those examined, whilst 90% had a negative result (a negative result indicates no pain; a positive result indicates the presence of pain). In contrast, for the right upper limb, 33% of those examined had a positive result, whilst 67% had a negative result. For all participants, several statistically significant associations were identified (Table 2). Sex-stratified analyses were considered exploratory because of the substantial imbalance in the number of women and men, particularly the small male subgroup (*n* = 10). In the group of men, only a statistically significant correlation was observed between the SOS questionnaire score and the Quick DASH questionnaire score (r = 0.6, *p* = 0.05), and between right-hand grip strength and left-hand grip strength (r = 0.9, *p* = <0.0001).

In the overall sample, higher SOS scores were associated with lower left-hand grip strength (ρ = −0.391, 95% CI −0.592 to −0.168; Holm-adjusted *p* = 0.001) and lower right-hand grip strength (ρ = −0.395, 95% CI −0.590 to −0.179; Holm-adjusted *p* = 0.001). Higher SOS scores were also associated with greater QuickDASH disability (ρ = 0.387, 95% CI 0.161 to 0.569; Holm-adjusted *p* = 0.001) and a positive right Finkelstein test (ρ = 0.294, 95% CI 0.068 to 0.493; Holm-adjusted *p* = 0.020), whereas the association with the left Finkelstein test was not statistically significant (ρ = −0.051; Holm-adjusted *p* = 0.645). NRS pain intensity was positively associated with QuickDASH score among participants with available NRS data (*n* = 42; ρ = 0.638, 95% CI 0.428 to 0.793; Holm-adjusted *p* < 0.001).

**Figure 1:**
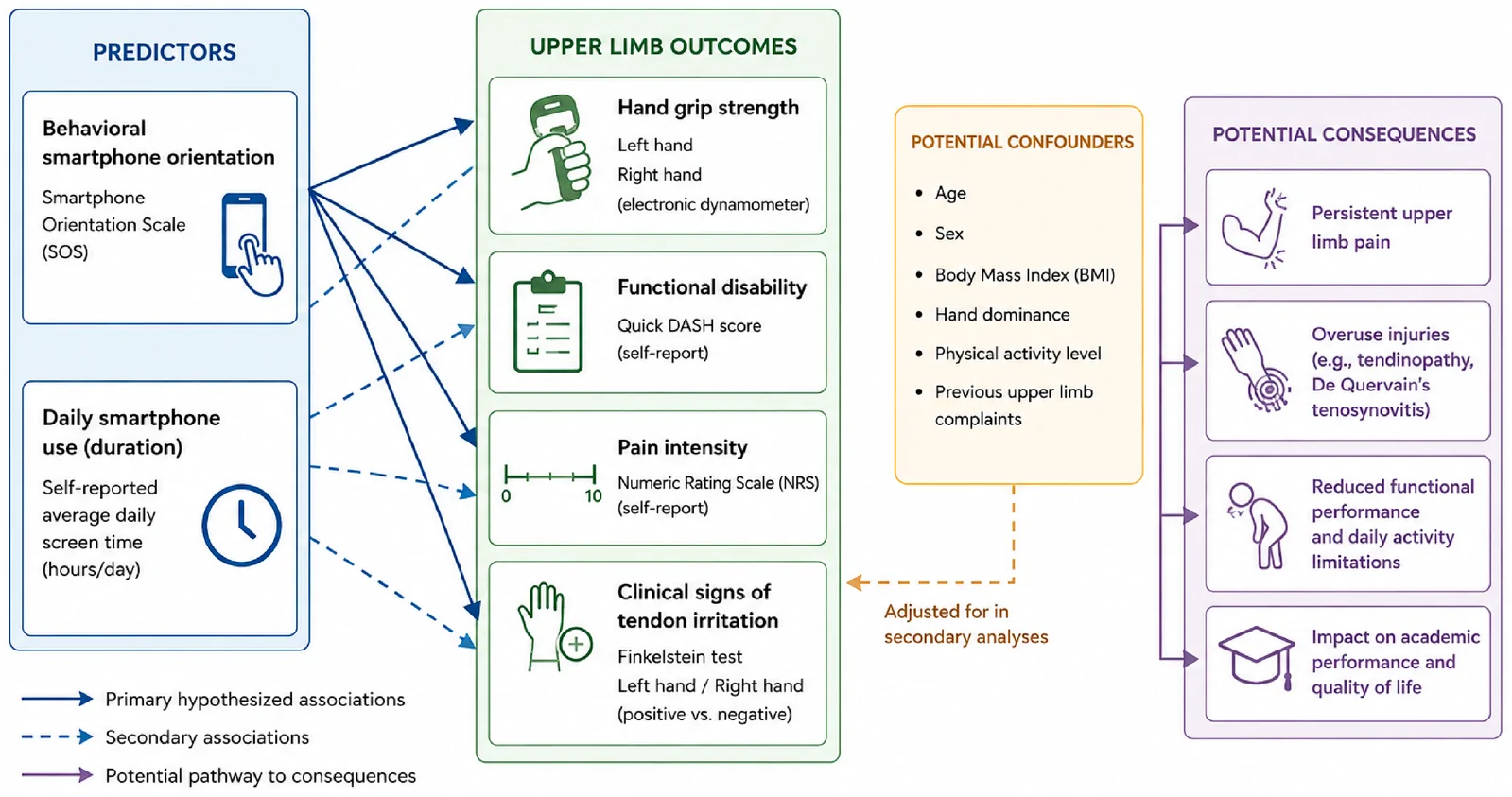
Conceptual model of the relationships between behavioral smartphone orientation, daily smartphone use, upper limb outcomes, potential confounders, and potential consequences.

Longer daily smartphone-use duration was associated with lower bilateral grip strength and higher SOS and QuickDASH scores in the overall sample after correction for multiple comparisons. No statistically significant association was observed between daily smartphone-use duration and correctly recalculated BMI. Sex-stratified analyses were considered exploratory owing to the small male subgroup (*n* = 10) and are presented in Supplementary Table S1. A conceptual model of the relationship relationships is presented below (Figure 1).

## 4. Discussion

The present study suggests that behavioral smartphone orientation may be more closely associated with objective and subjective markers of upper limb dysfunction than self-reported daily smartphone use. The results of our study suggest an association between daily smartphone use and upper limb function, grip strength and pain among university students. The results of our own study included 84 students from the Faculty of Health Sciences at the Pomeranian Medical University in Szczecin. Higher Smartphone Orientation Scale (SOS) scores were significantly associated with lower grip strength in both hands and higher QuickDASH scores and these associations remained statistically significant after Holm correction for multiple comparisons. A significant finding is that, despite a high level of smartphone orientation among 56% of the participants, only minor upper limb functional limitations were identified. This may suggest that, within this group of students, these findings may reflect relatively mild functional limitations in this young population, which requires further research and observation.

Similar results were reported by Kurtaran [16], who divided the participants into two groups based on their scores on the Smartphone Addiction Scale-short version (SAS-SV) questionnaire, identifying one group as smartphone-addicted and the other as non-addicted. The group of smartphone-addicted individuals scored an average of 14.2 on the Quick DASH questionnaire, whilst the average for the other group was 7 [16]. Din [17] also obtained similar results in his study, which likewise utilized the SAS-SV scale and the Quick DASH questionnaire. The study was conducted amongst individuals who used their smartphones for at least 4 h a day. The participants achieved an average score of 42 points on the SAS-SV questionnaire, which may indicate smartphone addiction. In the same group of young people, the average score on the Quick DASH questionnaire was 31, indicating a mild to moderate degree of upper limb functional impairment. Statistical analysis revealed a positive correlation between the SAS-SV score, indicating smartphone addiction, and the presence of upper limb disability [17].

Our study included 84 students and produced results that are consistent with the observations of other authors. The mean grip strength for the left hand was 22.1 ± 6.6 kg, and for the right hand, it was 22.6 ± 7.1 kg. Among men, the results were higher, with an average of 32.6 ± 4.8 kg for the left hand and 32.8 ± 4.9 kg for the right hand. In the group of women, the mean for the left hand was 20.7 ± 5.5 kg, and for the right hand, it was 21.3 ± 6.2 kg. The marked gender imbalance in the sample should be considered when interpreting the overall mean grip strength values. Hand grip strength was significantly associated with behavioral smartphone orientation. In the overall sample, higher SOS scores were associated with lower left-hand grip strength (ρ = −0.391, 95% CI −0.592 to −0.168; Holm-adjusted *p* = 0.001) and lower right-hand grip strength (ρ = −0.395, 95% CI −0.590 to −0.179; Holm-adjusted *p* = 0.001). Importantly, both associations remained statistically significant after correction for multiple comparisons. These findings indicate a moderate inverse association between behavioral smartphone orientation and bilateral hand grip strength; however, given the cross-sectional design, they should not be interpreted as evidence that smartphone use causes reduced grip strength. This indicates that higher smartphone orientation scores were associated with lower hand grip strength. In the study by Alshahrani et al. [18], the participants were all men. They were divided into two groups: one comprising individuals addicted to smartphones and the other comprising those who were not. In the group of smartphone-addicted individuals, the average grip strength for the dominant hand was 21.85 kg and for the non-dominant hand 20.05 kg. In the second group, the average grip strength for the dominant hand was 24.4 kg, and for the non-dominant hand, it was 21.35 kg. The results show that individuals addicted to smartphones have lower grip strength than those who are not addicted to smartphones [18]. Grip strength was also assessed by Din [17]. The results indicated a difference between the genders. The average for men was higher, at 45 kg, whereas for women the average was 23 kg. Statistical analysis revealed a negative correlation between smartphone addiction and grip strength (r = −0.208). This means that as the level of smartphone addiction increases, a decrease in grip strength was observed [17].

Pain in the upper limb is another focus of scientific research into the impact of smartphone use on the upper limb. 32% of respondents reported experiencing pain in the hand and/or wrist. Pain was most commonly felt in the right upper limb (76%), whereas 14% of students reported pain in the left upper limb. On the NRS scale, the average pain intensity score was 2.2 ± 1.8; for women, the average was higher at 2.3 ± 1.8. A positive result on the Finkelstein test was obtained by 10% of respondents for the left upper limb and by 33% for the right upper limb. Statistical analysis revealed a strong positive correlation between pain assessed on the NRS scale and the QuickDASH questionnaire score (*n* = 42; ρ = 0.638; 95% CI 0.428–0.793; Holm-adjusted *p* < 0.001). This finding indicates that higher pain intensity was associated with greater self-reported upper limb disability. Another significant finding of the study is the association between the SOS questionnaire score and the Finkelstein test result for the right upper limb (ρ = 0.294; 95% CI 0.068–0.493; Holm *p* = 0.020). A high level of smartphone use may involve more frequent and prolonged repetitive thumb movements, which could contribute to mechanical loading of the thumb tendons. However, a positive Finkelstein test alone is not sufficient to establish this diagnosis. This may lead to micro-injuries within the tendon sheaths of the extensor pollicis brevis and abductor pollicis longus muscles, as indicated by a positive Finkelstein test. Such findings may be associated with symptoms observed in de Quervain’s tenosynovitis, although a positive Finkelstein test alone is not sufficient to establish this diagnosis. The analysis indicated significant correlations between the severity of pain, the degree of functional limitation of the hand, a higher prevalence of a positive Finkelstein’s test result, and the level of smartphone use. Among the students studied by Salameh et al. [19], 34% of participants reported pain. Of those experiencing pain, 53% reported pain on the right side, whereas in 69% the pain was localized at the base of the thumb [19]. A similar issue concerning pain in the upper limb was analyzed by Baabdullah et al. [20]. Among the students surveyed, only 20.4% reported experiencing pain in the thumb or wrist area. Following the Finkelstein test, 19.1% of participants tested positive; of these, 10.8% were men and 28.4% were women [20].

Similar results were also reported in a study by Asad [15] on pain localized in the upper limb. Of the 200 participants, 60% experienced pain; of these, 101 participants tested positive on the Finkelstein test. The study showed that the prevalence of a positive Finkelstein test result increased with the number of text messages sent via a smartphone. When broken down by the number of messages sent, 40% of participants in the group sending up to 50 messages per day tested positive; in the group sending 50–100 text messages, the percentage was higher at 64%, whereas the highest figure of 80.9% was recorded in the group sending more than 200 messages per day [15]. The Finkelstein test was also used in the study by Nie [21]. A group of 937 students was recruited, of whom 52.1% tested positive on the Finkelstein test. An increase in positive Finkelstein test results was demonstrated as the time spent using smartphones increased. The average time spent using smartphones among those with a positive Finkelstein test result was 6.46 h per day. The figure among those without a positive result was lower, averaging 5.13 h per day [21].

Higher daily smartphone use was associated with less favorable upper-limb functional parameters, particularly among participants reporting longer daily use. These findings may provide a basis for future research examining whether smartphone orientation scores could be relevant to ergonomic education and physiotherapy-based prevention.

## 5. Study Limitations

This study has several limitations that should be taken into account when interpreting the results. The first of these is the relatively small sample size of students, all of whom were enrolled on medical degree programs, which significantly limits the extent to which the results can be generalized to the wider population. Given the sample size, the study had approximately 80% power to detect correlations of approximately |ρ| = 0.30. Consequently, weaker associations may not have been detected. Furthermore, there was a marked gender imbalance in the study group, with women constituting the vast majority (88%). This gender imbalance may be relevant when interpreting the results and generalizing them to the entire student population, in which men constituted a significantly smaller group. Furthermore, women rated their pain more highly on the NRS scale and scored higher on the Quick DASH questionnaire, which may indicate greater limitations in upper limb function within the group of women studied. Another limitation is the cross-sectional nature of the study, which restricts the ability to investigate cause and direct effect. This makes it impossible to conclusively state that smartphone use alone is the cause of upper limb functional impairment or the occurrence of pain. Further research is warranted in view of the constant development of technology and the resulting positive and negative effects on many aspects of mental, social and physical health.

The findings suggest that behavioral assessment using the Smartphone Orientation Scale may serve as a useful behavioral measure; however, its potential prognostic value for upper limb dysfunction remains to be validated in prospective studies. Incorporating ergonomic education and behavioral interventions into university health promotion programmers may therefore contribute to preventing cumulative musculoskeletal overload.

## 6. Conclusions

Despite frequent smartphone use, the majority of participants did not exhibit significant functional limitations, which may indicate an early stage of overuse. Pain was more commonly experienced in the dominant limb, which may indicate greater strain on that limb during smartphone use. As smartphone usage time increases, a deterioration in upper limb functional parameters is observed, particularly in groups with longer usage times. Ergonomic education and monitoring of smartphone use may be considered as potential preventive strategies, although their effectiveness requires further investigation.

In this cross-sectional study of university students, higher smartphone orientation scores were associated with indicators of upper limb dysfunction. These findings suggest that behavioral smartphone orientation may be associated with early manifestations of upper limb dysfunction among young adults. Future prospective studies are needed to further investigate these associations and determine whether behavioral patterns of smartphone use are related to the development or progression of upper limb dysfunction.

## Figures and Tables

**Table 1:** Descriptive characteristics of questionnaire scores and hand grip strength in the study population.

Parameters	Overall Mean ± SD		Women Mean ± SD		Men Mean ± SD		*p*-Value Women vs. Men	
NRS score	2.2 ± 1.8		2.3 ± 1.8		1 ± 1.2		0.1337	
SOS score	29.1 ± 8.8		29.1± 8.5		29.5 ± 11.2		0.8901	
QuickDASH score	15.7 ± 16.9		16.7 ± 17.2		8.2 ± 12.3		0.0262	
	**Overall** **Mean ± SD**		**Women** **Mean ± SD**		**Men** **Mean ± SD**		***p*** **-value** **women vs. men**	
	**Left**	**Right**	**Left**	**Right**	**Left**	**Right**	**Left**	**Right**
Hand grip strength (kg)	22.1 ± 6.6	22.6 ± 7.1	20.7 ± 5.5	21.3± 6.2	32.6 ± 4.8	32.8 ± 4.9	<0.001	<0.001

**Table 2:** Associations between smartphone orientation and upper limb outcomes in the overall study population.

Association	Group	*n*	ρ	95% CI	Raw *p*	Holm *p*
SOS score vs. left-hand grip strength	Overall	84	−0.391	−0.592 to −0.168	<0.001	0.001
SOS score vs. right-hand grip strength	Overall	84	−0.395	−0.590 to −0.179	<0.001	0.001
SOS score vs. QuickDASH score	Overall	84	0.387	0.161 to 0.569	<0.001	0.001
NRS pain score vs. QuickDASH score	Overall	42	0.638	0.428 to 0.793	<0.001	<0.001
SOS score vs. left Finkelstein test	Overall	84	−0.051	−0.261 to 0.182	0.645	0.645
SOS score vs. right Finkelstein test	Overall	84	0.294	0.068 to 0.493	0.007	0.020
Age at first smartphone use vs. SOS score	Overall	84	−0.246	−0.454 to −0.022	0.024	0.048
Left- vs. right-hand grip strength	Overall	84	0.966	0.940 to 0.976	<0.001	<0.001
SOS score vs. left-hand grip strength	Women	74	−0.507	−0.674 to −0.298	<0.001	<0.001
SOS score vs. right-hand grip strength	Women	74	−0.516	−0.681 to −0.312	<0.001	<0.001
SOS score vs. QuickDASH score	Women	74	0.377	0.134 to 0.589	<0.001	0.004
NRS pain score vs. QuickDASH score	Women	38	0.623	0.386 to 0.784	<0.001	<0.001
SOS score vs. left Finkelstein test	Women	74	−0.027	−0.258 to 0.248	0.819	0.819
SOS score vs. right Finkelstein test	Women	74	0.290	0.048 to 0.513	0.012	0.024
Age at first smartphone use vs. SOS score	Women	74	−0.353	−0.547 to −0.135	0.002	0.006
Left- vs. right-hand grip strength	Women	74	0.958	0.925 to 0.972	<0.001	<0.001
SOS score vs. left-hand grip strength	Men	10	−0.383	−0.775 to 0.281	0.275	1.000
SOS score vs. right-hand grip strength	Men	10	−0.103	−0.720 to 0.539	0.776	1.000
SOS score vs. QuickDASH score	Men	10	0.489	−0.222 to 0.845	0.151	0.908
NRS pain score vs. QuickDASH score	Men	4	0.943	0.816 to 1.000	0.057	0.400
SOS score vs. left Finkelstein test	Men	10	−0.291	−0.730 to 0.059	0.415	1.000
SOS score vs. right Finkelstein test	Men	10	0.321	−0.434 to 0.863	0.366	1.000
Age at first smartphone use vs. SOS score	Men	10	0.175	−0.591 to 0.810	0.629	1.000
Left- vs. right-hand grip strength	Men	10	0.794	0.173 to 1.000	0.006	0.049

Note: Finkelstein test was coded 0 = negative and 1 = positive. Confidence intervals are percentile bootstrap 95% CIs based on 5000 resamples. Holm correction was applied separately within each group across the eight associations shown.

## Data Availability

The original contributions presented in this study are included in the article/Supplementary Material. Further inquiries can be directed to the corresponding authors.

## Supplementary Materials

The following supporting information can be downloaded at: https://www.mdpi.com/article/10.3390/jcm15207857/s1, Table S1. Exploratory sex-stratified associations between daily smartphone use and study outcomes.

## Abbreviations

The following abbreviations are used in this manuscript:

NRS	Numeric Rating Scale
SOS	The Smartphone Orientation Scale
