# Supplementary material for: Behavioral Smartphone Orientation Is Associated with Upper Limb Dysfunction Among University Students: A Cross-Sectional Study
Source: J Clin Med. 2026 Oct 12;15(20):7857. doi: 10.3390/jcm15207857 (PMC13706254; doi:10.3390/jcm15207857)
Supplement: Supplementary file 1 [file jcm-15-07857-s001.zip › jcm-4572082-supplementary.pdf]

Table S1. Exploratory sex-stratified associations between daily smartphone use and study outcomes.

Association	Group	n	ρ	95% CI	Raw p	Holm p
Daily smartphone use vs. Left-hand grip strength	Overall	84	-0.307	-0.501 to -0.094	0.004	0.013
Daily smartphone use vs. Right-hand grip strength	Overall	84	-0.295	-0.489 to -0.090	0.006	0.013
Daily smartphone use vs. BMI (recalculated)	Overall	84	0.155	-0.067 to 0.366	0.159	0.159
Daily smartphone use vs. SOS score	Overall	84	0.455	0.246 to 0.625	<0.001	<0.001
Daily smartphone use vs. QuickDASH score	Overall	84	0.392	0.183 to 0.577	<0.001	<0.001
Daily smartphone use vs. Left-hand grip strength	Women	74	-0.396	-0.584 to -0.185	<0.001	0.002
Daily smartphone use vs. Right-hand grip strength	Women	74	-0.369	-0.556 to -0.151	0.001	0.003
Daily smartphone use vs. BMI (recalculated)	Women	74	0.157	-0.088 to 0.383	0.182	0.182
Daily smartphone use vs. SOS score	Women	74	0.483	0.278 to 0.655	<0.001	<0.001
Daily smartphone use vs. QuickDASH score	Women	74	0.380	0.163 to 0.575	<0.001	0.003
Daily smartphone use vs. Left-hand grip strength	Men	10	-0.006	-0.577 to 0.541	0.986	1.000
Daily smartphone use vs. Right-hand grip strength	Men	10	-0.095	-0.634 to 0.531	0.793	1.000
Daily smartphone use vs. BMI (recalculated)	Men	10	0.013	-0.842 to 0.661	0.972	1.000
Daily smartphone use vs. SOS score	Men	10	0.309	-0.600 to 0.923	0.385	1.000
Daily smartphone use vs. QuickDASH score	Men	10	0.499	-0.171 to 0.843	0.142	0.712

Note: Daily smartphone-use duration was coded ordinally from 1 (≤2 h/day) to 5 (>8 h/day). BMI was recalculated as weight (kg)/height (m)². Holm correction was applied separately within each group across the five associations shown.
